# Tailoring the Compositions and Nanostructures of Trimetallic Prussian Blue Analog‐Derived Carbides for Water Oxidation

**DOI:** 10.1002/advs.202402916

**Published:** 2024-09-03

**Authors:** Lujiao Mao, Jie Liu, Rong Lin, Jinhang Xue, Yuandong Yang, Shaojie Xu, Qipeng Li, Jinjie Qian

**Affiliations:** ^1^ Key Laboratory of Carbon Materials of Zhejiang Province College of Chemistry and Materials Engineering Wenzhou University Wenzhou Zhejiang 325035 P. R. China; ^2^ College of Chemistry and Chemical Engineering Zhaotong University Zhaotong Yunnan 657000 P. R. China

**Keywords:** carbon nanotube, competitive coordination, Mn_2_Co_2_C, oxygen evolution, Prussian blue analog

## Abstract

The electrochemical splitting of water for hydrogen production faces a major challenge due to its anodic oxygen evolution reaction (OER), necessitating research on the rational design and facile synthesis of OER catalysts to enhance catalytic activity and stability. This study proposes a ligand‐induced MOF‐on‐MOF approach to fabricate various trimetallic MnFeCo‐based Prussian blue analog (PBA) nanostructures. The addition of [Fe(CN)_6_]^3−^ transforms them from cuboids with protruding corners (MnFeCoPBA‐I) to core–shell configurations (MnFeCoPBA‐II), and finally to hollow structures (MnFeCoPBA‐III). After pyrolysis at 800 °C, they are converted into corresponding PBA‐derived carbon nanomaterials, featuring uniformly dispersed Mn_2_Co_2_C nanoparticles. A comparative analysis demonstrates that the Fe addition enhances catalytic activity, while Mn‐doped materials exhibit excellent stability. Specifically, the optimized MnFeCoNC‐I‐800 demonstrates outstanding OER performance in 1.0 m KOH solution, with an overpotential of 318 mV at 10 mA cm^−2^, maintaining stability for up to 150 h. Theoretical calculations elucidate synergistic interactions between Fe dopants and the Mn_2_Co_2_C matrix, reducing barriers for oxygen intermediates and improving intrinsic OER activity. These findings offer valuable insights into the structure‐morphology relationships of MOF precursors, advancing the development of highly active and stable MOF‐derived OER catalysts for practical applications.

## Graphical abstract

This study proposes a precise control over the carbon nanomaterials derived from a trimetallic PBA as electrocatalysts by unraveling the structure‐morphology‐activity relationship. The epitaxial grown material (MnFeCoNC‐I‐800) maintains structural and morphological stability after carbonization. Meanwhile, the enhanced electrocatalytic activity and stability of MnFeCoNC‐I‐800 are closely linked to the structural morphology and composition of materials, as evidenced by theoretical calculations.

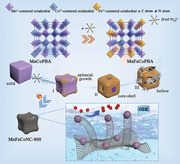

## 1 Introduction

As one of the green and sustainable energy sources, hydrogen energy can be readily generated by the electrochemical splitting of water.^[^ ^1^, ^2^, ^3^ ^]^ However, its anodic oxygen evolution reaction (OER) involves a multi‐step transfer process that greatly impedes the overall splitting efficiency.^[^ ^4^, ^5^ ^]^ In light of these challenges, conducting research on the design and synthesis of OER catalysts to improve the catalytic activity and stability holds paramount importance.^[^ ^6^, ^7^, ^8^ ^]^ This effort has the potential to lower the cost and energy consumption associated with hydrogen production, thereby promoting the advancement of efficient energy conversion and storage technologies. Although noble metal‐based OER electrocatalysts, such as RuO_2_ and IrO_2_, are considered ideal candidates, their high cost and poor stability under working conditions impose significant limitations on their large‐scale applications.^[^ ^9^, ^10^ ^]^ Recently, cost‐effective and abundant transition metal‐based catalysts, such as Fe─NC and Co─NC, have exhibited promising OER catalytic properties.^[^ ^11^, ^12^ ^]^ By judiciously selecting the metallic components of these OER catalysts, it becomes possible to effectively modulate the synergistic interactions among metals and precisely control their activity and stability.

On the other hand, the OER performance of nanomaterials is significantly influenced by their morphological features.^[^ ^13^, ^14^ ^]^ The synthesis of nanomaterials can yield a variety of forms, such as rods, tubes, sheets, cubes, and hollow nanostructures.^[^ ^15^, ^16^ ^]^ In general, nanocatalysts with different morphologies exhibit a larger surface area, showcasing superior surface OER activity owing to their higher density of active sites.^[^ ^17^, ^18^ ^]^ Additionally, the crystal structure orientation at the interface of OER catalysts can influence electronic transport. Diverse crystal structures and molecular arrangements lead to varying surface energies and interactions, thereby affecting the growth patterns and ultimate morphology of materials.^[^ ^19^, ^20^, ^21^ ^]^ Consequently, the selection and control of nanomaterial morphology and crystal structure becomes crucial for optimizing and controlling their performance.^[^ ^22^, ^23^, ^24^ ^]^ Moreover, a comprehensive understanding of the relationship between morphology and structure contributes to the precise design and synthesis of OER catalysts to meet practical applications.

Multifunctional metal‐organic frameworks (MOFs) have been demonstrated as excellent OER precursors on account of their tunable nanostructures and rich morphologies.^[^ ^25^, ^26^, ^27^ ^]^ To achieve a balance between MOF morphology control as well as their composition selection remains a significant challenge in materials science and engineering. Herein, we report a ligand‐induced competitive coordination method to synthesize the trimetallic **MnFeCoPBA‐I/II/III** series. By adding [Fe(CN)6]^3−^, the prefabricated MnCo‐based Prussian blue analog (**MnCoPBA**) cubes evolve from epitaxial growth to core‐shell structure and finally to hollow structure. Among them, the epitaxial grown materials maintain structural and morphological stability after carbonization, and the optimal **MnFeCoNC‐I‐800** shows excellent electrocatalytic OER performance with satisfactory long‐term stability. Theoretical calculations reveal the synergistic interactions between the dopant of Fe and the Mn_2_Co_2_C matrix, which significantly reduce the barriers for oxygen intermediates, thus improving the intrinsic activity for OER. This work involves precise tuning of the compositions and nanostructures of Fe‐doped Mn_2_Co_2_C as OER catalysts, which will shed light on the elucidation of the structure‐morphology‐activity relationship from MOF precursors.

## 2 Results and Discussion

**Figure 1:**
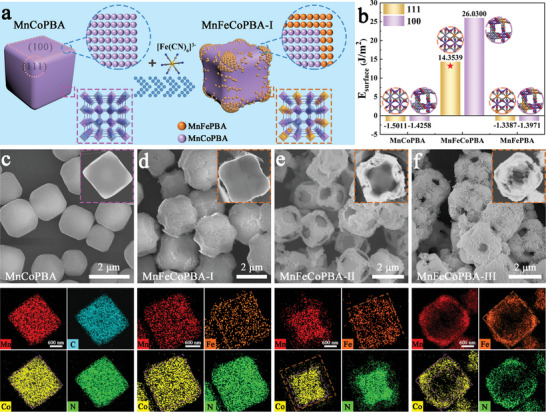
a) Schematic formation of trimetallic PBA heterostructures; b) Surface energies of MnCoPBA, MnFePBA and MnFeCoPBA for the (111)/(100) faces; SEM, TEM and the corresponding elemental mapping images of (c) MnCoPBA, (d) MnFeCoPBA‐I, (e) MnFeCoPBA‐II, and (f) MnFeCoPBA‐III, respectively.

Micro‐sized **MnCoPBA** precursors exhibit a cuboid morphology with a uniform distribution of Mn element throughout the material in **Figure** **1**c, Figures S1 and S2 (Supporting Information). Upon the introduction of external K_3_[Fe(CN)_6_], the facile generation of various trimetallic PBAs is accomplished through a competitive coordination method (Figure 1a).^[^ ^28^ ^]^ Owing to distinct bonding affinities with Mn(II) ions, diverse strengths of coordination interactions between [Fe(CN)_6_]^3−^ and [Co(CN)_6_]^3−^ emerge (Tables S1 and S2, Supporting Information). Utilizing this approach, three types of binary heterostructures are clearly discernible: a cuboid with protruding corners (**MnFeCoPBA‐I**), a core‐shell configuration (**MnFeCoPBA‐II**), and a hollow structure (**MnFeCoPBA‐III**) in Figure 1d–f, Figures S3–S6 (Supporting Information). Subsequently, theoretical calculations are conducted to ascertain the energy levels of crystals on the (100) and (111) facets (Figures S7 and S8, Supporting Information). The results reveal negative energy values for both crystal facets in MnCo‐/MnFe‐based PBAs, signifying the instability of both bimetallic structures (Figure 1b;Table S3, Supporting Information). Only when all three metals coexist does stability ensue, with the positive energy of the (111) facet (14.35 J m^−2^) being lower than that of the (100) facet (26.03 J m^−2^), suggesting heightened surface stability for the (111) plane of MnFeCo‐based PBA. Consequently, secondary growth initiates from the corners to generate a **MnFePBA** shell at the apex corner of the **MnCoPBA** crystal. At this juncture, the outer frame constructed by the stronger Mn─N≡C─Fe bonds continues to grow, while the core constituted by the weaker Mn─N≡C─Co bonds slowly dissolves, eventually yielding a well‐defined hollow nanostructure. These findings are further corroborated by energy‐dispersive X‐ray spectroscopy (EDX) analysis and elemental mapping in Figures S9–S12 and Table S4 (Supporting Information).

**Figure 2:**
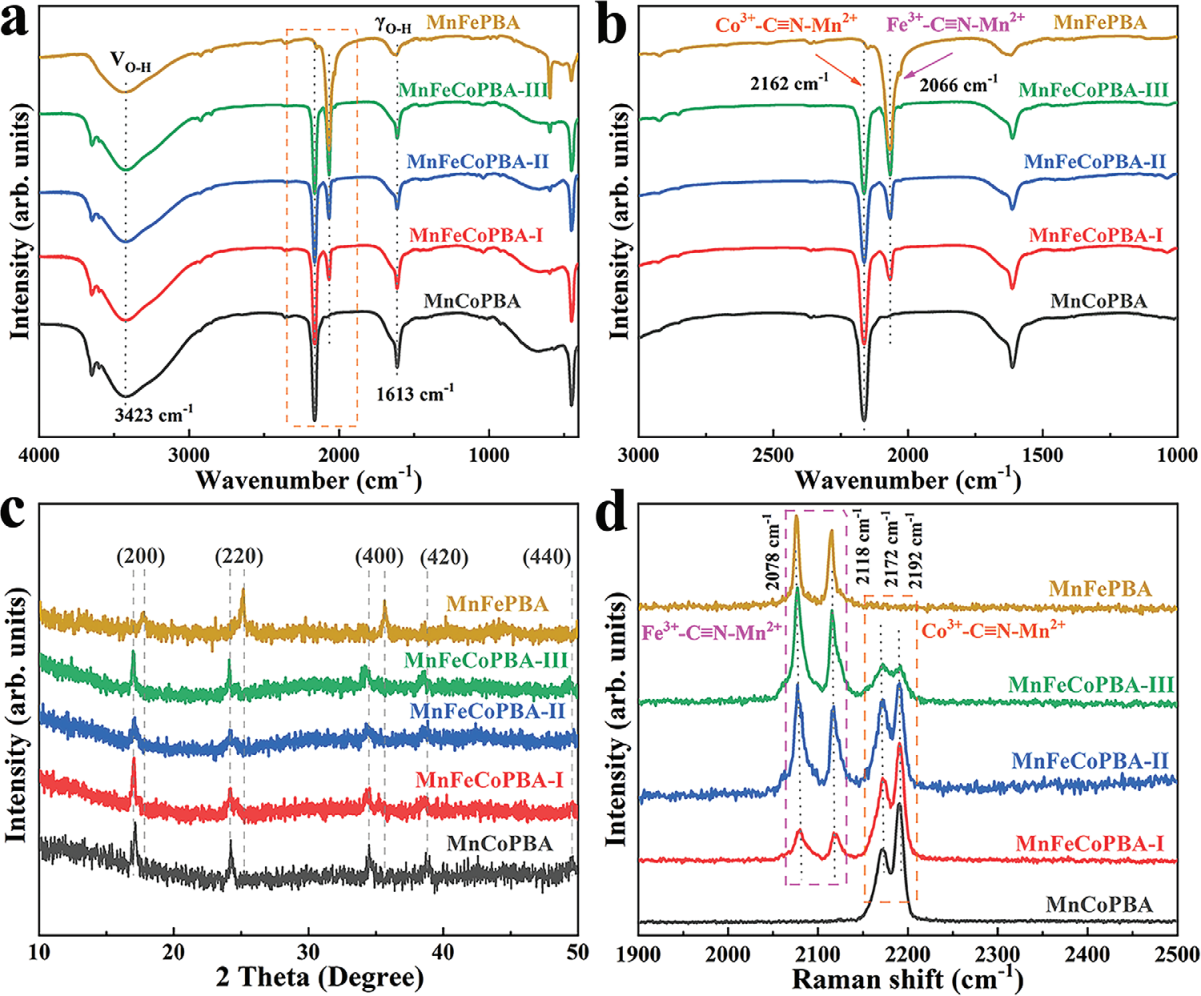
a,b) FT‐IR spectra, c) PXRD patterns, d) Raman spectra of MnCoPBA, MnFePBA, and MnFeCoPBA series.

A series of physicochemical characterizations have been performed to reveal the above structural and morphological transition. First, the Fourier transform infrared (FT‐IR) spectra display a characteristic γ_O‐H_ signal at 1613 cm^−1^ from lattice water, with the peak at 3423 cm^−1^ identified as the O─H stretching vibration, indicating the presence of protective water in the shell layer (**Figure** **2**a). The magnified regions in Figure 2b show two distinct peaks attributed to Co─C≡N─Mn (2162 cm^−1^) and Fe─C≡N─Mn (2066 cm^−1^). Significantly, the coexistence of both **MnCoPBA** and **MnFePBA** is evidenced by the doublet peaks. Its intensity of the vibrational peak of Fe─C≡N─Mn increases with reaction time, reflecting the cumulative growth of the MnFe‐based PBA layer. All **MnFeCoPBA** series closely correspond to pure MnCo‐based precursors with moderate crystallinity, and the diffraction peaks at 17.0°, 24.2°, 34.5°, 38.7°, and 49.4° in **MnFeCoPBA** are reasonably assigned to (200), (220), (400), (420), and (440) crystal planes, respectively (Figure 2c). The Raman spectra in Figure 2d exhibit characteristic peaks at 2078 and 2118 cm^−1^ arising from the Fe─C≡N─Mn bonds in **MnFeCoPBA** and **MnFePBA**, while peaks at 2172 and 2192 cm^−1^ originate from the Co─C≡N─Mn bonds in **MnCoPBA**.^[^ ^29^ ^]^ Concurrently, the peaks associated with Co─C≡N─Mn gradually weaken, consistent with the dissolution of the MnCo‐based core in **MnFeCoPBA‐III**. Furthermore, all desolvated samples manifest type I isotherms to indicate the presence of micropores with a main pore size of ≈0.5 nm (Figure S13, Supporting Information). In Table S5 (Supporting Information), the Brunauer‐Emmett‐Teller (BET) surface area values for **MnFeCoPBA‐I/II**/**III** are 464.9, 482.7, and 305.7 m^2^ g^−1^, respectively.

**Figure 3:**
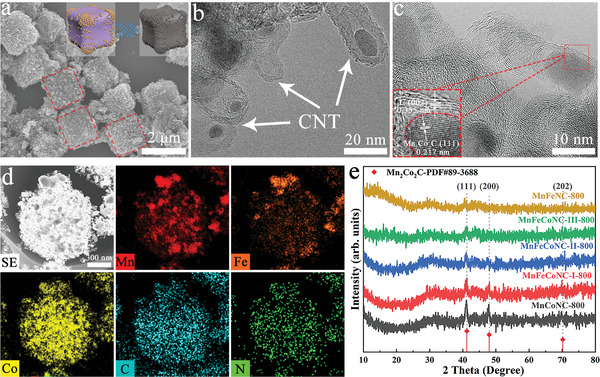
a) SEM, b) TEM, c) HAADF‐STEM images, d) the corresponding elemental mapping images of MnFeCoNC‐I‐800; e) PXRD patterns of PBA‐derived carbon nanocomposites.

Both bimetallic and trimetallic PBAs undergo a three‐stage weight‐loss process, with stabilization occurring at temperatures above 750°°C (Figure S14, Supporting Information). Consequently, these **MnFeCoPBA** series are subjected to pyrolysis at 800°°C, resulting in the formation of N‐doped carbon nanomaterials (**MnFeCoNC‐I/II/III‐800**). In **Figure** **3**a, the calcined samples maintain a cubic shape after carbonization without significant morphological deformation. The transmission electron microscopy (TEM) images exhibit the growth of abundant carbon nanotubes on the surface due to the catalytic effect of the metal particles (Figure 3b).^[^ ^30^ ^]^ High‐resolution TEM image exhibits lattice spacings of 0.217 and 0.335 nm for the (111) plane of Mn_2_Co_2_C and the (002) plane of graphitic carbon, respectively (Figure 3c). On the other hand, the elemental mapping images of **MnFeCoNC‐I‐800** verify the simultaneous presence of Mn, Fe, Co, C, and N elements in Figure 3d. Figure 3e presents three main peaks at 41.2°, 48.0°, and 70.2° belonging to the (111), (200), and (202) crystal faces of Mn_2_Co_2_C, respectively. Additional characterizations for control samples of **MnFeCoNC‐II/III‐800** are provided in Figures S15 and S16 (Supporting Information). In this context, the inner core of **FeMnCoNC‐II‐800** is predominantly preserved after carbonization, while the obtained **FeMnCoNC‐III‐800** experiences severe collapse due to the inherent instability of the empty shell. The Raman spectra own two distinct peaks corresponding to the defective sp^3^ carbon (D band, 1350 cm^−1^) and graphitic sp^2^ carbon (G band, 1580 cm^−1^) in Figure S17 (Supporting Information). Notably, **MnFeCoNC‐I‐800** manifests a lower I_D_/I_G_ ratio of 0.81 with a higher degree of graphitization, which can significantly enhance the electron transfer kinetics during electrocatalysis. Finally, the nitrogen isotherms, pore size distribution (PSD curves, and specific surface area values of these carbon materials are comprehensively described in Figure S18 and Table S5 (Supporting Information).

**Figure 4:**
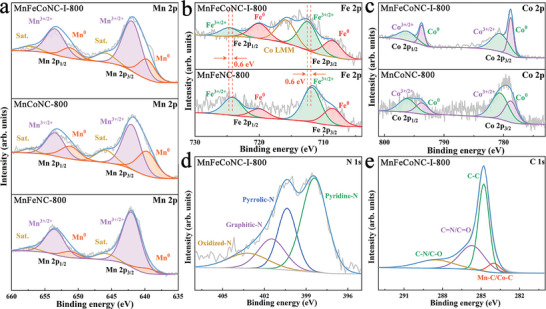
High‐resolution XPS spectra of MnFeCoNC‐I‐800, MnFeNC‐800 and MnCoNC‐800 for a) Mn 2p, b) Fe 2p, c) Co 2p, d) N 1s and e) C 1s.

The resulting carbon composites are subjected to X‐ray photoelectron spectroscopy (XPS) to investigate their chemical environment, with **MnFeNC‐800** and **MnCoNC‐800** serving as comparison samples. The full survey spectra affirm the presence of Mn, Co, Fe, C, N, and O in all pyrolyzed nanomaterials, as depicted in Figure S19 (Supporting Information). High‐resolution Mn 2p spectra reveal metallic Mn(0) at 639.7 and 651.2 eV, alongside dominant Mn(II) 2p_3/2_ and 2p_1/2_ signals at 641.9 and 653.5 eV, respectively, with satellite peaks at 645.9 and 657.3 eV (**Figure** **4**a). In Figure 4b, the deconvoluted Fe 2p spectra display two metallic Fe(0) peaks at 708.6/720.0 eV, and two more Fe(II/III) peaks at 712.4/724.7 eV. However, the distribution area of Fe in **MnFeCoNC‐I‐800** is notably smaller than that of **MnFeNC‐800**, indicating a trace amount of Fe. In this case, a slight positive shift of 0.6 eV is observed for **MnFeCoNC‐I‐800** that results from electron transfer among multiple metals, potentially improving the electrocatalytic activity.^[^ ^31^ ^]^ For Co 2p, two sharp peaks at 778.8 and 793.7 eV were assigned to Co─C and Co─Co bonds in **MnFeCoNC‐I‐800** (Figure 4c), consistent with Mn_2_Co_2_C structure. Meanwhile, a pair of peaks at 780.6 and 796.2 eV are attributed to Co(II/III) 2p_3/2_ and 2p_1/2_, respectively. The N 1s spectra exhibit five subpeaks at 398.4, 400.4, 401.4, and 403.2 eV, corresponding to pyridinic‐N, pyrrolic‐N, graphitic‐N, and oxidized‐N, respectively (Figure 4d). A higher concentration of graphitic and pyridinic nitrogen species is reported to enhance the OER.^[^ ^32^, ^33^ ^]^ The deconvoluted C 1s spectra in Figure 4e display peaks at 284.0, 284.8, 285.7, and 288.5 eV, attributed to Mn─C/Co─C, C─C, C═N/C═O, and C─N/C─O in **MnFeCoNC‐I‐800,** respectively. Peaks for Mn─C/Co─C and Mn─N/Co─N bonds are observed in the C 1s and N 1s spectra of **MnCoNC‐800**, but not detected in **MnFeNC‐800** (Figures S20 and S21, Supporting Information).

**Figure 5:**
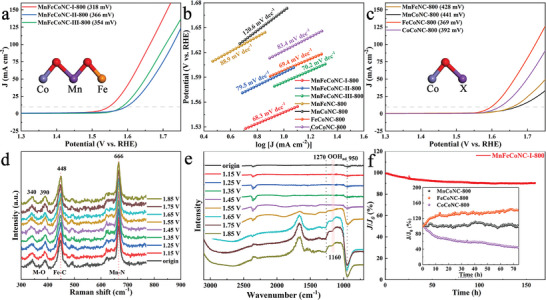
a, c) LSV curves of MnFeCoNC‐I/II/III‐800, MnFeNC‐800, MnCoNC‐800, FeCoNC‐800 and CoCoNC‐800; b) The corresponding Tafel plots; d) In situ Raman spectra; e) In situ FT‐IR spectra; f) OER stability test for MnFeCoNC‐I‐800, MnCoNC‐800, FeCoNC‐800 and CoCoNC‐800.

The electrochemical performance of PBA‐derived carbon nanomaterials as OER catalysts is evaluated within a three‐electrode system. Among them, **MnFeCoNC‐I‐800** exhibits the lowest overpotential (η_10_ = 318 mV) at 10 mA cm^−2^, outperforming **MnFeCoNC‐III‐800** (354 mV) and **MnFeCoNC‐II‐800** (366 mV) in **Figure** **5**a. This performance is competitive with similar reported electrocatalysts in Tables S6 and S7 (Supporting Information). In Figures S22 and S23 (Supporting Information), the optimal **MnFeCoNC‐I‐800** obviously exhibits a lower onset potential compared to RuO_2_. A comparative analysis of this series of bimetallic catalysts reveals relatively inferior η_10_ values in Figure 5c **MnFeNC‐800**, 428 mV; **MnCoNC‐800**, 441 mV; **FeCoNC‐800**, 369 mV; and **CoCoNC‐800**, 392 mV. The synergistic effect is more pronounced in the optimized trimetallic system, where the introduction of Fe ions effectively improves the catalyst activity. Additionally, **MnFeCoNC‐I‐800** owns a small Tafel slope of 68.3 mV dec^−1^, with the lowest charge transfer resistance (40.9 Ω, Figure 5b; Figure S24, Supporting Information). The electrochemically active surface area is further evaluated by calculating the double‐layer capacitance (C_dl_) to show good linearity (Figure S25, Supporting Information). **MnFeCoNC‐I‐800** has a C_dl_ value of 16.5 mF cm^−2^, much larger than that of **MnFeCoNC‐II‐800** (14.2 mF cm^−2^), **MnFeNC‐800** (6.7 mF cm^−2^), and other comparison samples in Figure S26 (Supporting Information). Furthermore, the enhanced Raman peaks manifest four peaks at 340/390, 448, and 666 cm^−1^ ascribed to M─O, Fe─C, and Mn─N vibration at 1.15–1.85 V, while the active substance is not obviously decomposed (Figure 5d). In Figure 5e, the weak signal of the OOH intermediate is detected in the in situ FT‐IR spectra at 1160 cm^−1^. It is evident that the monometallic catalyst is extremely unstable, whereas the bimetallic catalyst may transform into active species due to the existence of Fe ions.^[^ ^34^ ^]^ However, the prepared electrode of **MnCoNC‐I‐800** demonstrates satisfactory stability with minimal decay after 2000 cycles, benefiting from the presence of Mn ions (Figure 5f; Figure S27 and Table S8, Supporting Information).^[^ ^35^ ^]^ This clearly indicates a four‐electron process with almost 100% Faraday efficiency, suggesting that the oxidation current originates from intrinsic oxygen evolution (Figures S28 and S29, Supporting Information). After the stability test, the phase of Mn_2_Co_2_C retains, and scanning electron microscopy (SEM) images reveal a relatively intact morphology (Figure S30, Supporting Information). Compared to pristine **MnFeCoNC‐I‐800**, post‐test **MnFeCoNC‐I‐800** exhibits an oxidized surface in Figure S31 (Supporting Information), and a clear cubic structure is observed, directly indicating that the cube‐like morphology has very good firmness. Lastly, the shape and position of the XPS spectra in Figure S32 (Supporting Information) do not obviously alter, except for a slight change in the intensity of the peak.

**Figure 6:**
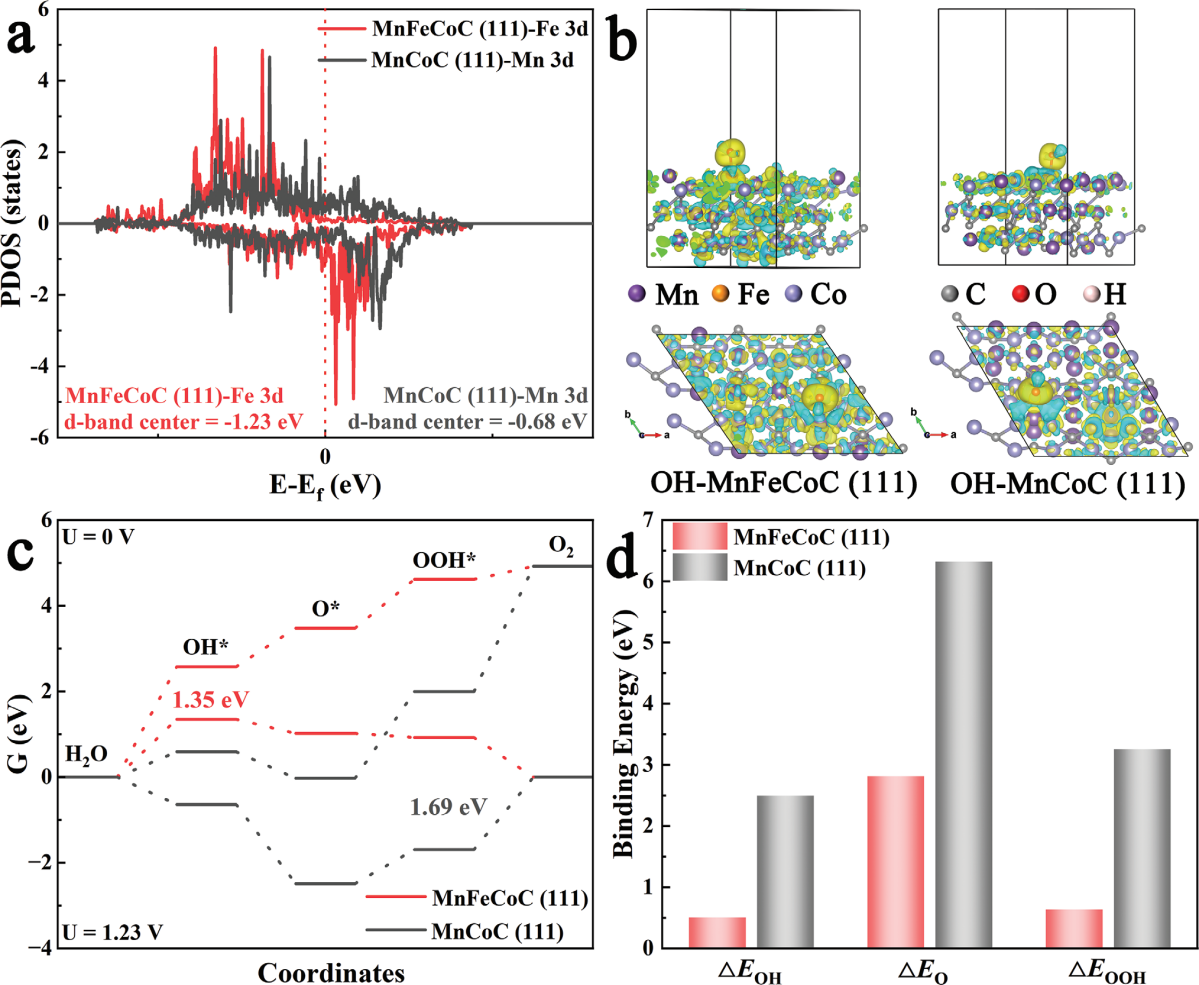
a) The PDOS plots of MnFeCoC(111) and MnCoC(111); b) The charge density difference from top and side views of OH─MnFeCoC(111) and OH─MnCoC(111) (yellow/cyan isosurfaces represent the charge accumulation/depletion); c) Free energy diagram of OER; d) Binding energy of the OER intermediates.

To further elucidate the enhanced OER activity and stability, two models of **MnCoC(111)** and **MnFeCoC(111)** are reasonably constructed to mimic the Mn_2_Co_2_C and its Fe‐doped counterpart, respectively (Figures S33 and S34, Supporting Information). The calculated projected density of states (PDOS) plots reveal that the introduction of Fe altered the active site of **MnCoC(111)** in **Figure** **6**a. Compared to the Mn 3d orbital, the 3d orbital of Fe exhibits a greater occupancy in the anti‐bonding state, causing a shift in the center of the d band away from the Fermi level. This implies a weakened binding affinity toward oxygen species.^[^ ^36^ ^]^ In Figure 6b, the differential charge diagram shows a strengthened electron transfer between atoms at the interface within **MnFeCoC(111)**. Meanwhile, a consistent upward trend is observed without the applied potential for **MnFeCoC(111)**, indicating each step as an endothermic reaction (Figure 6c). At the equilibrium potential (U = 1.23 V), the adsorption of OH* and the desorption of OOH* emerge as the rate‐determining steps, with energy barriers of 1.35 and 1.69 eV, respectively. This alteration suggests that the introduction of Fe significantly modifies the adsorption capacity of O species, and the lower energy barrier facilitates the occurrence of OER on **MnFeCoC(111)**. Compared to **MnCoC(111)**, the binding energy diagram of OER intermediates in Figure 6d illustrates a decrease in OH, O, and OOH binding energies from 2.49/6.31/3.25 to 0.50/2.81/0.63 eV for **MnFeCoC(111)**. According to Sabatier's principle, the excessive binding energy of oxygen intermediates is unfavorable for the 4‐electron reaction of OER.^[^ ^37^ ^]^ Therefore, the introduction of Fe by competitive coordination method aids in regulating the strong adsorption of oxygen species by Fe‐doped Mn_2_Co_2_C, ultimately enhancing OER performance.

## 3 Conclusion

In this study, we propose a facile hydrothermal synthesis strategy to fabricate a variety of trimetallic **MnFeCoPBA** nanomaterials with various shapes, facilitated by ligand‐induced competitive coordination. Upon pyrolysis, **MnFeCoNC‐I‐800** retains its original cubic morphology, while core‐shell and hollow‐structured control samples collapse due to structural instability. The calcined **MnFeCoNC‐I‐800** exhibits a high specific surface area, high graphitization, and abundant carbon nanotubes, showcasing low overpotential (η_10_ = 318 mV) and satisfactory stability at 10 mA cm^−2^ during OER. The enhanced electrocatalytic activity and stability of **MnFeCoNC‐I‐800** are closely linked to the structural morphology and composition of materials, as evidenced by theoretical calculations. It is revealed that the introduction of Fe effectively regulates the sorption capacity of oxygen intermediates by doping Fe into Mn_2_Co_2_C. This study provides guidance for constructing pristine MOFs and MOF‐derived nanostructures with diverse morphologies, offering valuable insights into developing catalysts with high activity and stability for practical oxygen evolution.

## Conflict of Interest

The authors declare no conflict of interest.

## Author Contributions

L.M., J.L., and R.L. equally contributed to this work. Methodology, data curation, formal analysis, investigation, validation, theoretical calculation, and writing‐original draft were performed by L.M. Methodology, data curation, formal analysis, validation, theoretical calculation, and writing‐original draft were performed by J.L. Data curation and formal analysis were performed by R.J., J.X., Y.Y., and S.X. Conceptualization, formal analysis, investigation, and supervision were performed by Q.L. Conceptualization, formal analysis, funding acquisition, investigation, project administration, and supervision were performed by J.Q.

## Supporting information

Supporting Information

## Data Availability

The data that support the findings of this study are available from the corresponding author upon reasonable request.
