# Supplementary material for: Tailoring the Compositions and Nanostructures of Trimetallic Prussian Blue Analog‐Derived Carbides for Water Oxidation
Source: Adv Sci (Weinh). 2024 Sep 3;11(42):2402916. doi: 10.1002/advs.202402916 (PMC11558108; doi:10.1002/advs.202402916)
Supplement: Supplementary file 1 — Supporting Information [file ADVS-11-2402916-s001.docx]

**Supporting Information**

# Tailoring the compositions and nanostructures of trimetallic Prussian blue analogue-derived carbides for water oxidation

Lujiao Mao,^a,#^ Jie Liu,^a,#^ Rong Lin,^a,#^ Jinhang Xue,^a,^ Yuandong Yang,^a,^ Shaojie Xu,^a,^ Qipeng Li,^b,^* and Jinjie Qian^a,^*

^a^Key Laboratory of Carbon Materials of Zhejiang Province, College of Chemistry and Materials Engineering, Wenzhou University, Wenzhou 325035, Zhejiang, P. R. China

^b^College of Chemistry and Chemical Engineering, Zhaotong University, Zhaotong 657000, Yunnan, P. R. China

^#^These authors contributed equally to this work.

*Corresponding author

E-mail: qpli@ztu.edu.cn (Q. Li); jinjieqian@wzu.edu.cn (J. Qian)

**Number of pages: 52 ( S1-S52)**

**Number of tables: 8 ( Table S1-Table S8)**

**Number of figures: 34 ( Figure S1- Figure S34)**

**Table of contents**

**Experimental Section S3**

**Structural Details S9**

**Additional Images S10**

**Gas Sorption Data S21**

**Additional Electrochemical Data S30**

**Additional Tables S43**

**References S51**

**Experimental Section**

**Chemicals and Reagents**

All chemicals are purchased and used without further purification. Potassium hexacyanocobaltate (III) (K_3_[Co(CN)_6_], 99%, Aladdin), potassium ferricyanide (III) (K_3_[Fe(CN)_6_], 99%, Aladdin), manganese acetate tetrahydrate (Mn(CH_3_COO)_2_∙4H_2_O, AR, 99%, Aladdin), Sodium dodecyl sulfate (CH_3_(CH_2_)_11_OSO_3_Na, SDS, ACS, ≥99%, Aladdin), potassium hydroxide (KOH AR, 90%, Aladdin), Nafion solution (5 wt%, Dupont), Ethanol (EtOH, ≥99.5%, Aladdin) and deionized water (DI H_2_O, 18 MΩ). All the salts, solvents and other reagents are of analytical grade. High-purity N_2_, Ar gases, and deionized water are used in all experiments.

**Synthesis of MnCoPBA**

In a 35.0 mL pressure tube, a mixture of Mn(CH_3_COO)_2_∙4H_2_O (80 mg) and SDS (300 mg) was added to 3 mL H_2_O and stirred to give a homogeneous solution. Besides, 132 mg K_3_[Co(CN)_6_] was added to 5 mL H_2_O and dissolved to obtain solution A. After that, solution A was injected into the tube to shake well and stand for 15 min. Finally, after washing it with H_2_O and EtOH and drying, the white powder of **MnCoPBA** was obtained successfully.

**Synthesis of MnFeCoPBA**

22.0 mg of K_3_[Fe(CN)_6_] was dissolved in 0.5 mL of H_2_O to obtain A solution. In addition, A solution was added to the **MnCoPBA** solution which was left to stand for 15 min. Then solution was placed at 70 °C for 30 min/2h/4h. Under room temperature, the product was successfully collected after washing three times with H_2_O and three times with EtOH, then dried in a vacuum oven at 85 °C, denoted as **MnFeCoPBA-I/II/III**. Based on the used organic ligand, the yield of **MnFeCoPBA-I** is calculated to be about 46.1%, and the repeatability is excellent.

**Synthesis of MnFeCoNC-I/II/III-800**

The synthesized **MnFeCoNC-I/II/III** samples were placed on the quartz tubes, and further placed in a CVD tube furnace for heat treatment. Under an atmosphere of Ar (150 sccm), the temperature was heated to 800 °C at a heating rate of 10 °C min^-1^ for 2 hours. After carbonization at 800 °C, MnFeCoNC-800 series can maintain about 49.4% of precursor mass.

**Material Characterization**

The samples are characterized by using scanning electron microscopy (SEM) on a JEOL JSM 6700F microscope, transmission electron microscopy (TEM) on an FEI Tecnai F20 electron microscope, high-resolution transmission electron microscope (HR-TEM) and energy dispersive X-ray spectroscopy (EDS) analyses are carried out under JEOL JEM-2100F microscope operating at an accelerating voltage of 200 kV. And powder X-ray diffraction (PXRD) (D/Max2000, Rigaku) using a Bruker D8-Advance powder diffractometer operates at 40 kV, 40 mA for Cu Kα radiation (λ = 1.5406 Å). Ramanspectrometer (LabRAM HR Evolution) is used to investigate the phase composition of samples at GS1000. Raman spectrometer (LabRAM HR Evolution) is used to investigate the phase composition of samples at GS1000. Fourier transform infrared spectroscopy (FT-IR) spectra are carried on in the model of PerkinElmer Frontier MIR. Thermal gravimetric analysis (TGA) is performed on a NETZSCH STA 449C instrument where pure nitrogen as a carrier gas with a heating rate of 10 ^o^C min^-1^. X-ray photoelectron spectroscopy (XPS) is conducted on a Thermo Scientific ESCALAB 250. N_2_ sorption analysis is measured using the Specific Surface Area & Pore Size Analyzer (BSD-PS1 by BSD Instrument), after the samples are activated under 100 ^o^C they were placed into a clean ultra-high vacuum system and measured at the cryogenic temperature of 77 K. The specific surface areas are calculated by using the Brunauer-Emmett-Teller (BET) equation from the nitrogen adsorption data in the relative range (P/P_0_) of 0.04-0.2. The pore size distribution (PSD) plots are obtained from the adsorption and desorption branch of the isotherm based on the Barrett-Joyner-Halenda (BJH) model and/or Horvath-Kawazoe (H-K) models.

**Electrochemical measurements**

The electrochemical experiments are carried out at room temperature using a CHI 760E electrochemical station for OER. The experiment used the electrodes of Hg/Hg_2_Cl_2_ and platinum net are sequentially behaved as the reference (RE) and counter electrodes (CE), which are used in 1.0 M KOH electrolyte solution. According to a formula E_RHE_ = E_SCE_ + 0.242 V + 0.059 V × pH = E_SCE_ + 1.05, all of its potentials can be corrected to reversible hydrogen electrode (RHE) potentials. In a mixture solution of 500 μL of 3:2 v/v distilled water/ethanol, and 5 wt % Nafionsolution (30 μL), the catalyst powder (5 mg) is dispersed to form a homogeneous catalyst ink after 2 h’ sonication. Thereafter, the surface of glassy carbon (diameter: 3 mm) is loaded with 10 μL of a catalyst ink, wherein 1.3 mg cm^-2^ is calculated the loading amount. Linear sweep voltammetry (LSV) is conducted in electrolyte solution without correction (a scan rate: 5 mV s^-1^). The Tafel slope is transferred according to Tafel equation as follows: η = b · log(j / j_0_). Regarding the evaluation of the electrochemical active surface areas (ECSA) of the samples, CV has also been performed by measuring the double-layer capacitances (C_dl_) with various scan rate (20, 40, 60, 80 and 120 mV s^-1^) under the potential window of 1.04-1.16 V *vs.* RHE. Under the constant voltage, the EIS is performed to test the range of from 100 kHz to 0.01 Hz. For evaluating the long-term performance, the electrochemical stability of the catalyst is conducted at a constant overpotential for achieving a high initial current density. In order to calculate the electron transfer number, RRDE voltammogram of **MnFeCoNC-I/II/III-800** is conducted to collect. Faradaic efficiency, the ring potential is held constantly at 0.40 V *vs.* RHE to reduce the emerged O_2_ on the disk electrode at a rotation rate of 1600 rpm in N_2_-saturated 1.0 M KOH. Oxygen is generated by OER with a constant current on the disk electrode. Faradaic efficiency can be calculated by the collection efficiency of disk current, ring current and disc-ring electrode. In this system, all electrochemical tests are performed without iR-corrected.

**Surface energies calculations details**

Our calculations on the unit cell of MnCoPBA/MnFePBA, MnCoPBA/MnFePBA/MnFeCoPBA (100) surface and MnCoPBA/MnFePBA/MnFeCoPBA (111) surface were calculated by performing density functional theory (DFT) calculations using the CASTEP module of Accelrys Materials Studio 8.0 software to obtain the geometry optimizations structure with minimized energy. The pristine crystal structure and cell parameters of MnCoPBA were from the reported literature. The cleaved slabs from MnCoPBA/MnFePBA/MnFeCoPBA crystal structure were (100) surface and (111) surface. The generalized gradient approximation (GGA) with the Perdew-Burke-Ernzerhof (PBE) functional is used to treat the electronic exchange and correlation. In this computation, the MnCoPBA/MnFePBA/MnFeCoPBA is modeled in a 2×2×1 supercell with a vacuum space of 15 Å was used in the normal direction of the surface, which is thick enough for the system to converge to an accurate total energy. According to the following equation, we could obtain the corresponding surface energies of each crystal plane:

E_surface_ = (E_slab_ - nE_bulk_)/2A

where E_surface_ is the surface energy of the corresponding crystal plane, E_slab_ is the total energy of the surface slab, E_bulk_ is the total energy of the bulk unit cell, n is the ratio of the slab to the number of atoms contained in the bulk unit cells and A is the unit area of the surface.

**DFT calculations details**

**Density functional theory**

The density functional theory was used to carry out all the calculations with the Perdew-Burke-Ernzerh exchange-correlation functional of generalized gradient approximation and the projector-augmented wave method, which was implemented through Vienna Ab-initio Simulation Package (VASP).The input model structure file was created by VESTA.**^[1]^** The plane wave-basis expansion cutoff energy was fixed at 500 eV, and atomic relaxation was conducted until the force exerted on atoms was less than 0.02 eV Å^-1^ and energy was concurrently converged to 1×10^-5^ eV. The k-point was a Monkhorst-Pack of 3×3×1.

The Gibbs free energy change (ΔG) of each lithiation step was defined as:

ΔG = ΔE + ΔZPE - TΔS,

where ΔE is the electronic energy difference directly obtained from DFT calculations, ΔZPE is the change in zero-point energy, T is the temperature (T = 298.15 K) and ΔS is the change in the entropy, respectively.**^[2]^** The zero-point energy and entropy were obtained through vibrational frequencies.

Conventionally, in an alkaline electrolyte, the anode reactions after oxygen adsorption can be written as:

H_2_O ↔ OH* + H^+^ + e^–^ (1)

OH*↔O* + H^+^ + e^–^ (2)

O*+H_2_O ↔OOH* + H^+^ + e^–^ (3)

OOH*↔ O_2_* + H^+^ + e^–^ (4)

**Figure S1.** The crystal structures of **MnCoPBA** and **MnFeCoPBA**.

**Figure S2.** SEM images of **MnCoPBA**.

**Figure S3.** SEM images of **MnFeCoPBA-I**.

**Figure S4.** SEM images of **MnFeCoPBA-II**.

**Figure S5.** SEM images of **MnFeCoPBA-III**.

**Figure S6.** TEM images of (a) **MnCoPBA**, (b) **MnFeCoPBA-I**, (c) **MnFeCoPBA-II**, and (d) **MnFeCoPBA-III**, respectively.

The TEM images exhibited a distinct light-dark contrast, suggesting that the core and shell layers had different chemical compositions, a finding corroborated by the compositional line profile results (please see **Figures S10-S12** below).

**Figure S7.** The optimized models for surface energies of **MnCoPBA**, **MnFeCoPBA** and **MnFePBA** along the the (100) face.

**Figure S8.** The optimized models for surface energies of **MnCoPBA**, **MnFeCoPBA** and **MnFePBA** along the the (111) face.


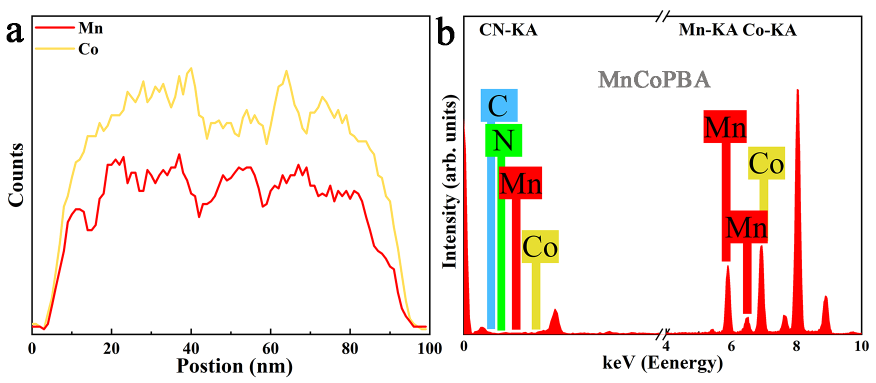


**Figure S9.** (a) the compositional line profile and (b) EDX element analysis of **MnCoPBA**.

**Figure S10.** (a) the compositional line profile and (b) EDX element analysis of **MnFeCoPBA-I**.

**Figure S11.** (a) the compositional line profile and (b) EDX element analysis of **MnFeCoPBA-II**.

**Figure S12.** (a) the compositional line profile and (b) EDX element analysis of **MnFeCoPBA-III**.

**Figure S13.** (a) N_2_ sorption isotherms, (b) The corresponding PSD curves of **MnCoPBA**, **MnFeCoPBA-I/II**/**III** and **MnFePBA**.

**Figure S14.** TGA curves of **MnCoPBA** and **MnFeCoPBA-I**.

**Figure S15**. (a-d) TEM image, HR-TEM image, EDX curve, HAADF-STEM image and element mappings of **MnFeCoNC-II-800**.

**Figure S16**. (a-d) SEM image, HR-TEM image, EDX curve, HAADF-STEM image and element mappings of **MnFeCoNC-III-800**.

**Figure S17.** Raman spectra of **MnCoNC-800**, **MnFeCoNC-I/II/III-800** and **MnFeNC-800**.

**Figure S18.** (a) N_2_ sorption isotherms, (b) The corresponding PSD curves of **MnCoNC-800**, **MnFeCoNC-I-800**, **MnFeCoNC-II-800**, **MnFeCoNC-III-800** and **MnFeNC-800**.

**Figure S19.** Full survey XPS spectra of **MnCoNC-800**, **MnFeCoNC-I/II/III-800** and **MnFeNC-800**.

**Figure S20**. The deconvoluted spectra of (a) C 1s; (b) N 1s of **MnCoNC-800**.

**Figure S21**. The deconvoluted spectra of (a) C 1s; (b) N 1s of **MnFeNC-800**.

**Figure S22.** (a) LSV curves and (b) Tafel plots of **MnFeCoNC-I/II/III-800** and RuO_2_.

**Figure S23.** Comparison of the overpotential at 10 mA cm^-2^ and Tafel slopes with the materials in this work.

**Figure S24.** EIS diagrams of (a) **MnFeCoNC-I-800**, **MnFeCoNC-II-800**, **MnFeCoNC-III-800**, **MnCoNC-800**, **MnFeNC-800**, (b) **FeCoNC-800** and **CoCoNC-800** in 1.0 M KOH.

The equivalent electrical circuit for EIS data (R1: solution resistance; R2 and CPE1: resistance and CPE impedance of electrical double layer at the interface of activated product layer and GCE substrate).

**Figure S25.** CV curves from 20 to 120 mV s^-1^ of (a) **MnFeCoNC-I-800**, (b) **MnFeCoNC-II-800**, (c) **MnFeCoNC-III-800**, (d) **MnCoNC-800**, (e) **MnFeNC-800** (f) **FeCoNC-800** and (g) **CoCoNC-800** in 1.0 M KOH.

**Figure S26.** C_dl_ curves under different scan rates of (a) **MnFeCoNC-I-800**, **MnFeCoNC-II-800**, **MnFeCoNC-III-800**, **MnCoNC-800**, **MnFeNC-800**, (b) **FeCoNC-800** and **CoCoNC-800** in 1.0 M KOH.

**Figure S27.** LSV curves of **MnFeCoNC-I-800** before and after 2000 cycles.

**Figure S28.** The corresponding electron transfer numbers of (a) **MnFeCoNC-I-800**, (b) **MnFeCoNC-II-800**, (c) **MnFeCoNC-III-800**.

**Figure S29.** Ring current of **MnFeCoNC-I-800**.

**Figure S30.** SEM images of **MnFeCoNC-I-800** (a) before and (b) after OER, and (c) PXRD patterns before and after OER.

**Figure S31.** (a) TEM and (b, c) HR-TEM images of **MnFeCoNC-I-800** after OER.

**Figure S32.** (a) The full survey spectra and high-resolution XPS spectra of (b) Co 2p, (c) Fe 2p, (d) Mn 2p for **MnFeCoNC-I-800** before and after OER.


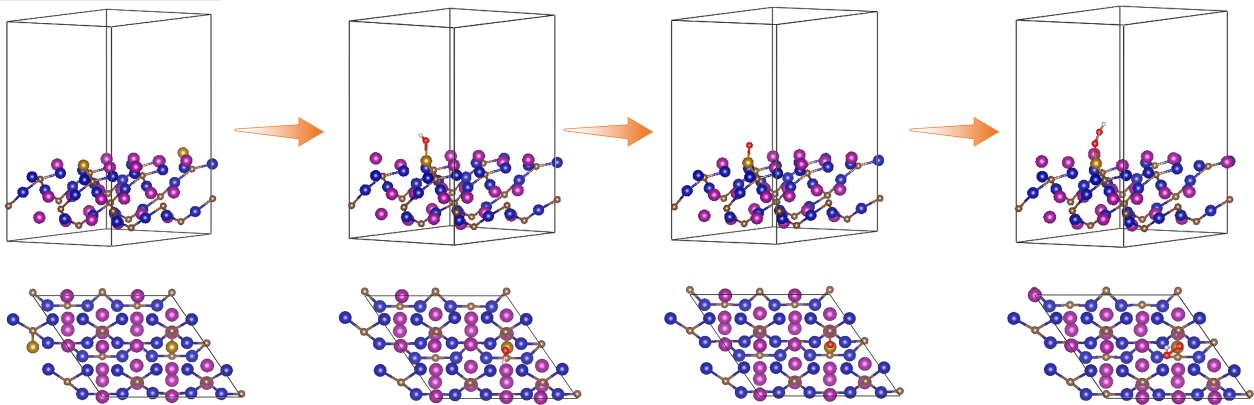


**Figure S33.** Top view and side view of the optimized configurations of oxygen intermediates (OH*, O*, and OOH*) adsorbed on **MnFeCoC (111)**.

**Figure S34.** Top view and side view of the optimized configurations of oxygen intermediates (OH*, O*, and OOH*) adsorbed on **MnCoC (111)**.

**Table S1. Summary of Crystal Data for MnCoPBA**.

| **Items** | **MnCoPBA^[3]^** |
| --- | --- |
| COD | 1521295 |
| Formula | Mn_3_[Co(CN)_6_]_2_ |
| Mass | 594.68 |
| crystal system | Cubic |
| space group | *Fmm* |
| *a* (Å) | 10.41 |
| *b* (Å) | 10.41 |
| *c* (Å) | 10.41 |
| *α* (°) | 90 |
| *β* (°) | 90 |
| *γ* (°) | 90 |
| *V* (Å^3^) | 1129.09 |

**Table S2. Summary of Crystal Data for MnFePBA**.

| **Items** | **MnFePBA^[4]^** |
| --- | --- |
| ICSD | 240929 |
| Formula | Mn_3_[Fe(CN)_6_]_2_ |
| Mass | 588.54 |
| crystal system | Cubic |
| space group | *Fmm* |
| *a* (Å) | 10.38 |
| *b* (Å) | 10.38 |
| *c* (Å) | 10.38 |
| *α* (°) | 90 |
| *β* (°) | 90 |
| *γ* (°) | 90 |
| *V* (Å^3^) | 1118.39 |

**Table S3. Surface Energies of MnCoPBA, MnFeCoPBA, and MnFePBA on the (100) and (111) Planes.**

| **Samples/Planes** | **(100)** | **S_(100)_** | **(111)** | **S_(111)_** | **bulk** |
| --- | --- | --- | --- | --- | --- |
| **MnCoPBA** | -1.4258 | 51.7958 | -1.5011 | 44.8565 | -10914.3002 |
| **MnFeCoPBA** | 26.0296 |  | 14.3539 |  | -10736.8451 |
| **MnFePBA** | -1.3971 |  | -1.3387 |  | -10204.4651 |

**Table S4. Mn/Fe/Co Ratio in Reactants and Quantitative Analyses of the Mn/Fe/Co Ratio in MnFeCoNC-X Samples by ICP Analysis.**

| **Samples** | Mn/Fe/Co ratio in reactants | **Sample element content** | | |
| --- | --- | --- | --- | --- |
|  |  | **Mn** | **Fe** | **Co** |
| **MnCoNC** | 1/0/1.2 | 26.791% | - | 24.505% |
| **MnFeCoNC-I-800** | 1/0.2/1.2 | 21.779% | 2.931% | 15.695% |
| **MnFeCoNC-II-800** |  | 22.327% | 4.941% | 18.358% |
| **MnFeCoNC-III-800** |  | 26.407% | 5.043% | 18.696% |

**Table S5. N_2_ Sorption Data.**

| **Samples** | **Surface area (m^2^ g^-1^)** | | **Total pore volume^a^ (cm^3^ g^-1^)** | **Micropore volume^b^ (cm^3^ g^-1^)** |
| --- | --- | --- | --- | --- |
|  | **BET method** | **Langmuir method** |  |  |
| **MnCoPBA** | 681.9812 | 1021.9244 | 0.4891 | 0.3421 |
| **MnFeCoPBA-I** | 464.9441 | 709.0194 | 0.4792 | 0.2315 |
| **MnFeCoPBA-II** | 482.6591 | 719.0988 | 0.5386 | 0.2342 |
| **MnFeCoPBA-III** | 305.7440 | 456.9085 | 0.1868 | 0.1529 |
| **MnFePBA** | 106.1156 | 168.8646 | 0.4901 | 0.0481 |
| **MnCoNC-800** | 56.8372 | 92.7898 | 0.5281 | 0.0225 |
| **MnFeCoNC-I-800** | 67.4209 | 111.0460 | 0.5072 | 0.0204 |
| **MnFeCoNC-II-800** | 48.5200 | 86.4411 | 0.2826 | 0.0179 |
| **MnFeCoNC-III-800** | 63.2565 | 116.7496 | 0.3477 | 0.0211 |
| **MnFe****NC-800** | 9.9702 | 17.2262 | 0.1416 | 0.0023 |

^a^ At P/P_0_= 0.99. ^b^ Determined by NLDFT method.

**Table S6. Electrochemical Parameters of** **MnFeCoNC-I-800, MnFeCoNC-II-800, MnFeCoNC-III-800, MnCoNC-800 and MnFeNC-800.**

| **Sample** | **Ƞ_10_ (mV)** | **Tafel slope**  **(mV dec^-1^)** | **C_dl_**  **(mF cm^-2^)** | **R_ct_**  **(Ω)** |
| --- | --- | --- | --- | --- |
| **MnFeCoNC-I****-800** | 318 | 68.3 | 16.5 | 40.9 |
| **MnFeCoNC-II-800** | 366 | 79.5 | 14.2 | 66.2 |
| **MnFeCoNC-III-800** | 354 | 70.2 | 23.3 | 63.4 |
| **MnFeNC-800** | 428 | 88.9 | 6.7 | 95.2 |
| **MnCoNC-800** | 441 | 120.6 | 13.4 | 90.5 |
| **FeCoNC-800** | 369 | 69.4 | 14.7 | >3000 |
| **CoCoNC-800** | 392 | 83.4 | 1.5 | >4000 |

**Table S7. OER Performance Comparison between MnFeCoNC-I-800 and Other Electrode Materials.**

| **Sample** | **Electrolyte** | **Ƞ_10_ (mV)** | **Tafel slope**  **(mV dec^-1^)** | **Ref.** |
| --- | --- | --- | --- | --- |
| **MnFeCoNC-I-800** | 1.0 M KOH | 318 | 68.3 | **This work** |
| **Ar-U-CoFe PBA** | 1.0 M KOH | 305 | 36.1 | **Ref 5** |
| **Fe-Co-Mn PBA** | 1.0 M KOH | 310 | 98 | **Ref 6** |
| **Ni_0.42_Co_0.58_F_2_-G** | 1.0 M KOH | 313 | 42.5 | **Ref 7** |
| **CoMo-MI-600** | 1.0 M KOH | 316 | 89.9 | **Ref 8** |
| **Ni_SA_-O/Mo_2_C** | 1.0 M KOH | 299 | 89.36 | **Ref 9** |
| **MOF-Fe/Co(1:1)** | 1.0 M KOH | 321 | 84 | **Ref 10** |
| **NiPc-NiFe_0.05_** | 1.0 M KOH | 333 | 58 | **Ref 11** |
| **NiMn-MOFs+MCCF** | 1.0 M KOH | 320 | 93 | **Ref 12** |
| **MCCF/NiMn-MOFs** | 1.0 M KOH | 280 | 86 |  |
| **FeCo_2_O_4_@FeCo_2_S_4_@PPY** | 1.0 M KOH | 360 | 65.1 | **Ref 13** |
| **CF/Ni_3_N/VON** | 1.0 M KOH | 287 | 120.9 | **Ref 14** |
| **Co_2.4_Ni_0.6_Ge_2_O_5_(OH)_4_** | 1.0 M KOH | 340 | 59.8 | **Ref 15** |

**Table S8. Stability of OER Performance Comparison between MnFeCoNC-I-800 and Other Transition Metal-Based Electrocatalysts.**

| **Sample** | **Electrolyte** | **Stability at 10 mA cm^-2^ (hours)** | **Ref.** |
| --- | --- | --- | --- |
| **MnFeCoNC-I-800** | 1.0 M KOH | 160 | **This work** |
| **Ni_0.42_Co_0.58_F_2_-G** | 1.0 M KOH | 130 | **Ref 7** |
| **Ni_SA_-O/Mo_2_C** | 1.0 M KOH | 16 | **Ref 9** |
| **NiFe-MOF/G** | 1.0 M KOH | 32 | **Ref 16** |
| **Ar-U-CoFe PBA** | 1.0 M KOH | 20 | **Ref 17** |
| **CoNiFeO_x_-NC** | 1.0 M KOH | 40 | **Ref 18** |
| **H-2D Co/Mo_2_C@NC** | 1.0 M KOH | 10 | **Ref 19** |
| **NiFe MOF** | 1.0 M KOH | 24 | **Ref 20** |

**References:**

[1] K. Momma, F. Izumi, *J. Appl. Cryst.* 2011, **44**, 1272-1276.

[2] J. Cao, X. Zhang, S. Zhao, X. Lu, H. Ma, *Phys. Chem. Chem. Phys.* 2022, **24**, 21030-21039.

[3] G. Małecki, A. Ratuszna, *Power Diffr.* 1999, **14**, 25-30.

[4] J.-Q. Li, F.-C. Zhou, Y.-H. Sun, J.-M. Nan, *J. Alloys Compd.* 2018, **740**, 346-354.

[5] F. Diao, M. Rykær Kraglund, H. Cao, X. Yan, P. Liu, C. Engelbrekt, X. Xiao, *J. Energy Chem.* 2023, **78**, 476-486.

[6] Q. Zhang, H. Wang, W. Han, L. Yang, Y. Zhang, Z. Bai, *Nano Research* 2023, **16**, 3695-3702.

[7] Z. Xu, W. Zuo, Y. Yu, J. Liu, G. Cheng, P. Zhao, *Adv. Sci.* 2024, **11**, 2306758.

[8] Y. Guo, Q. Huang, J. Ding, L. Zhong, T.-T. Li, J. Pan, Y. Hu, J. Qian, S. Huang, *Int. J. Hydrogen Energy* 2021, **46**, 22268-22276.

[9] M. Hou, L. Zheng, D. Zhao, X. Tan, W. Feng, J. Fu, T. Wei, M. Cao, J. Zhang, C. Chen, *Nat. Commun.* 2024, **15**, 1342.

[10] K. Ge, S. Sun, Y. Zhao, K. Yang, S. Wang, Z. Zhang, J. Cao, Y. Yang, Y. Zhang, M. Pan, *Angew. Chem., Int. Ed.* 2021, **60**, 12097-12102.

[11] J. Li, P. Liu, J. Mao, J. Yan, W. Song, *J. Mater. Chem. A* 2021, **9**, 11248-11254.

[12] W. Cheng, X. Lu, D. Luan, X. Lou, *Angew. Chem., Int. Ed.* 2020, **59**, 18234-18239.

[13] D. Zhao, M. Dai, Y. Zhao, H. Liu, Y. Liu, X. Wu, *Nano Energy* 2020, **72**, 104715.

[14] T. Xiong, J. Li, J. Chandra Roy, M. Koroma, Z. Zhu, H. Yang, L. Zhang, T. Ouyang, M.S. Balogun, M, *J. Energy Chem.* 2023, **81**, 71-81.

[15] B. Yang, N. Zhang, G. Chen, K. Liu, J. Yang, A. Pan, M. Liu, X. Liu, R. Ma, T. Qiu, *Appl. Catal. B: Environ.* 2020, **260**, 118184.

[16] Y. Wang, B. Liu, X. Shen, H. Arandiyan, T. Zhao, Y. Li, M. Garbrecht, Z. Su, L. Han, A. Tricoli, C. Zhao, *Adv. Energy Mater.* 2021, **11**, 2003759.

[17] F. Diao, M. Rykær Kraglund, H. Cao, X. Yan, P. Liu, C. Engelbrekt, X. Xiao, *J. Energy Chem.* 2023, **78**, 476-486.

[18] C. Chen, Y. Tuo, Q. Lu, H. Lu, S. Zhang, Y. Zhou, J. Zhang, Z. Liu, Z. Kang, X. Feng, D. Chen, *Appl. Catal. B: Environ.* 2021, **287**, 119953.

[19] C. Hyoun Ahn, W. Seok Yang, J. Jae Kim, G. Sudha Priyanga, T. Thomas, N.G. Deshpande, H. Seong Lee, H. Koun Cho, Chem. Eng. J. 2022, 435, 134815.

[20] Y. Liu, X. Li, Q. Sun, Z. Wang, W.-H. Huang, X. Guo, Z. Fan, R. Ye, Y. Zhu, C.-C. Chueh, C.-L. Chen, Z. Zhu, *Small* 2022, **18**, 2201076.
